# Myeloid Cell-Derived IL-1 Signaling Damps Neuregulin-1 from Fibroblasts to Suppress Colitis-Induced Early Repair of the Intestinal Epithelium

**DOI:** 10.3390/ijms25084469

**Published:** 2024-04-18

**Authors:** Ding Qiu, Shaoting Xu, Kaile Ji, Ce Tang

**Affiliations:** 1 Department of Gastroenterology and Hepatology, The First Affiliated Hospital, Sun Yat-sen University, No.58, Zhong Shan Er Lu, Guangzhou 510080, China; qiud5@mail2.sysu.edu.cn; 2 Institute of Precision Medicine, The First Affiliated Hospital, Sun Yat-sen University, Guangzhou 510080, China; xushaotingst@163.com (S.X.); jikle@mail2.sysu.edu.cn (K.J.); 3 Animal Experiment Center, The First Affiliated Hospital, Sun Yat-sen University, Guangzhou 510080, China

**Keywords:** neuregulin-1, fibroblasts, ulcerative colitis, inflammation, IL-1β

## Abstract

Neuregulin-1 (Nrg1, gene symbol: *Nrg1*), a ligand of the ErbB receptor family, promotes intestinal epithelial cell proliferation and repair. However, the dynamics and accurate derivation of Nrg1 expression during colitis remain unclear. By analyzing the public single-cell RNA-sequencing datasets and employing a dextran sulfate sodium (DSS)-induced colitis model, we investigated the cell source of *Nrg1* expression and its potential regulator in the process of epithelial healing. Nrg1 was majorly expressed in stem-like fibroblasts arising early in mouse colon after DSS administration, and Nrg1–Erbb3 signaling was identified as a potential mediator of interaction between stem-like fibroblasts and colonic epithelial cells. During the ongoing colitis phase, a significant infiltration of macrophages and neutrophils secreting IL-1β emerged, accompanied by the rise in stem-like fibroblasts that co-expressed Nrg1 and IL-1 receptor 1. By stimulating intestinal or lung fibroblasts with IL-1β in the context of inflammation, we observed a downregulation of *Nrg1* expression. Patients with inflammatory bowel disease also exhibited an increase in *NRG1*^+^*IL1R1*^+^ fibroblasts and an interaction of NRG1–ERBB between *IL1R1*^+^ fibroblasts and colonic epithelial cells. This study reveals a novel potential mechanism for mucosal healing after inflammation-induced epithelial injury, in which inflammatory myeloid cell-derived IL-1β suppresses the early regeneration of intestinal tissue by interfering with the secretion of reparative neuregulin-1 by stem-like fibroblasts.

## 1. Introduction

Ulcerative colitis (UC) is a type of chronic inflammatory bowel disease (IBD) characterized by unremitting inflammation of the colon and rectum. Patients experience a range of debilitating symptoms including diarrhea, abdominal pain, rectal bleeding, and weight loss [1,2]. Current understanding suggests that UC arises from a combination of environmental triggers in genetically susceptible individuals [2]. Defects in the epithelial barrier, dysregulated immune responses, and microbial imbalances are all key contributors to the initiation and persistence of inflammation [2,3]. To address the damage to the intestinal surface barrier resulting from uncontrolled immune response, the epithelial cells rapidly undergo resealing, while migrating to provide coverage for the wound [4]. Following the closure of the wound defect, they further mature and differentiate to restore numerous functions of the mucosa. However, the process of mucosal healing is intricate and generally influenced by various molecules and cellular components within the microenvironment. Immune cells, particularly macrophages and neutrophils, hinder the healing of intestinal mucosa in UC development by releasing pro-inflammatory cytokines [5,6]. Such a cytokine storm activates fibroblasts, which significantly influence the inflammatory response, tissue remodeling, and ultimately, colonic fibrosis [7,8].

Neuregulin-1 (mouse gene symbol: *Nrg1*; human: *NRG1*), a member of the epidermal growth factor (EGF) family, regulates diverse cellular processes through ErbB receptor activation and downstream signaling cascades, impacting cell growth, differentiation, and survival [9,10]. Nrg1 is essential for nervous system development, axon and limb regeneration, post-spinal cord injury repair, and myocardial cell proliferation [11,12,13,14]. Notably, Nrg1 expression is upregulated in macrophages and fibroblasts of UC patients [15]. Furthermore, studies suggest that mesenchymal-derived Nrg1 plays a crucial role in intestinal mucosal repair following radiation injury [16]. However, the precise spatiotemporal dynamics of Nrg1 expression during UC development and progression remain unclear.

Interleukin-1 receptor type 1 (mouse gene symbol: *Il1r1*; human: *IL1R1*), a transmembrane receptor for IL-1α and β, is expressed on various cell types, including both immune and non-immune cells [8,17,18]. While the roles of IL-1 in immune cell response, such as Th17 cell differentiation and γδT cell activation, have been elucidated [18], the accurate kinetics and potential function(s) of IL-1 signaling in intestinal tissue cells in the context of colitis, however, are still obscure.

Single-cell RNA-sequencing (scRNA-seq) analysis is widely implemented to clarify tissue ecosystems under healthy or pathological conditions [19]. Compared to bulk RNA-seq, which only obtains the average expression of genes, scRNA-seq enables us to conduct a more in-depth and intuitive analysis of the alteration of target cells and molecules. Through this technology, the gene profile of each single colon tissue or immune cell, the detailed taxonomy of each intestine-associated cell type, and the potential interactions between distinct cell subsets involved in the process of inflammation or intestinal repair can be explored at a much higher resolution.

In this study, we identified a distinct subpopulation of fibroblasts co-expressing neuregulin-1 and IL-1R1 by reanalyzing the public scRNA-seq datasets generated previously by other researchers and flow cytometry analysis. By in vivo and in vitro experiments, we confirmed the importance of IL-1 signaling in suppressing Nrg1 expression in intestinal fibroblasts. We hypothesize that these fibroblasts play a crucial role in epithelial proliferation and wound healing during the early stages of colitis. Inflammatory myeloid cells likely act as a regulator of these *Nrg1*^+^*Il1r1*^+^ fibroblasts by releasing IL-1 signaling to suppress neuregulin-1-mediated intestinal epithelial repair and exacerbate inflammation. Our study provides a potential therapeutic strategy for IBD treatment by facilitating intestinal mucosal repair by targeting the IL-1–IL-1R–neuregulin-1 axis.

## 2. Results

### 2.1. Composition and Kinetics of Colonic Cells during the Process of Mouse Colitis

**Figure 1:**
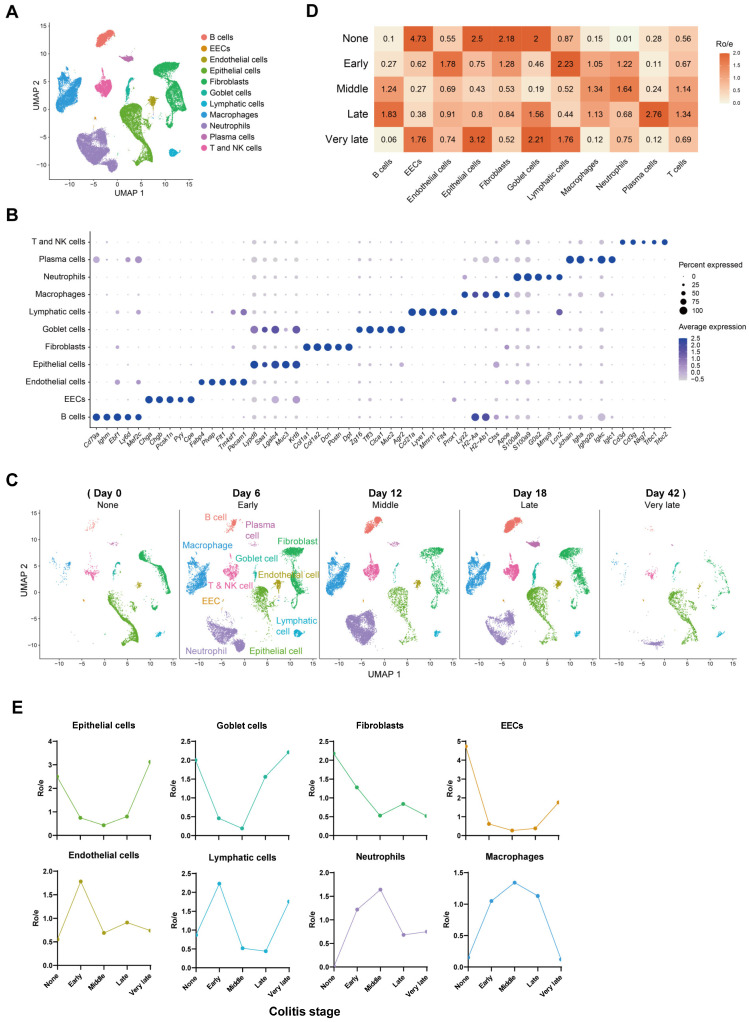
**Single-cell RNA-Seq reveals murine mucosal cell types during epithelial healing after DSS-induced colitis.** (**A**) UMAP projection of 45,014 high-quality cells colored by cell type. (**B**) Bubble plot depicting expression levels of selected marker genes for each cell type. (**C**) UMAP projection of different cell types in each stage of epithelium healing. (**D**) Estimated tissue prevalence of each cell type using the Ro/e score. (**E**) Kinetics of Ro/e of intestinal tissue cells and myeloid immune cells.

To elucidate cellular contributions to mucosal healing in DSS-induced colitis, we obtained single-cell RNA-sequencing (scRNA-seq) data of murine acute colitis from the GEO public database (GSE193342) [20]. After data integration and quality control, 45,014 cells were analyzed. Cell clustering and identification using established markers revealed 11 distinct cell types: B cells (*Cd79a*, *Ighm*, *Ebf1*), enteroendocrine cells (EECs, *Chga*, *Chgb*, *Pyy*), endothelial cells (*Pecam1*, *Plvap*, *Flt1*), epithelial cells (*Muc3*, *Krt8*, *Saa1*), fibroblasts (*Col1a1*, *Col1a2*, *Dcn*), goblet cells (*Zg16*, *Muc2*, *Tff3*), lymphatic cells (*Ccl21a*, *Lyve1*, *Prox1*), macrophages (*Lyz2*, *H2-Aa*, *H2-Ab1*), neutrophils (*S100a8*, *S100a9*, *G0s2*), plasma cells (*Jchain*, *Igha*, *Igkc*), and T and NK cells (*Cd3d*, *Trbc1*, *Nkg7*) (Figure 1A,B). We further assessed the distribution and dynamics of these cell types across disease stages (Figure 1C). Early colitis initiation was characterized by increased endothelial cells and infiltration of inflammatory lymphatic cells, neutrophils, and macrophages (Figure 1D,E). Conversely, epithelial cells, goblet cells, fibroblasts, and EECs displayed a significant decrease upon exposure to acute inflammation (Figure 1D,E). In the late and very late stages, inflammation subsided, with progressive restoration of epithelial and goblet cells. However, fibroblast levels did not fully recover, suggesting potential permanent loss of some intestinal stromal cells during inflammation.

### 2.2. Accumulation of Colonic Nrg1-Expressing Fibroblasts in the Acute Colitis Phase

**Figure 2:**
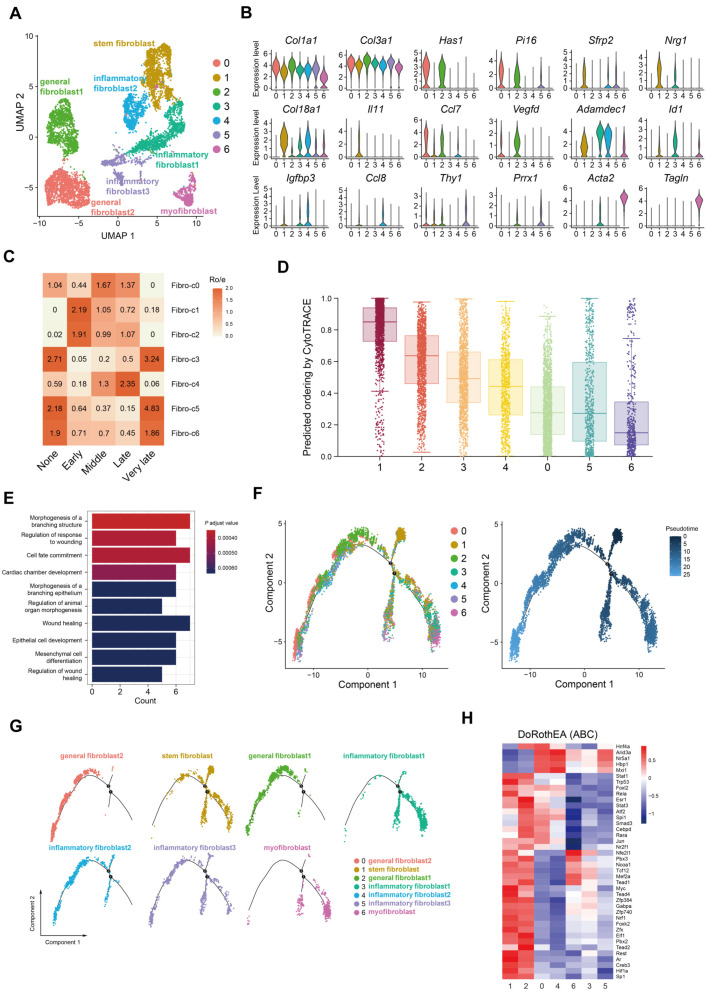
**Early enrichment of *Nrg1*^+^ fibroblasts associated with epithelial healing.** (**A**) UMAP projection of 7572 fibroblasts colored by cluster. (**B**) Violin plots showing expression levels of marker genes across fibroblast subclusters. (**C**) Estimated tissue prevalence of each fibroblast subcluster using the Ro/e score. (**D**) Boxplot depicting the differentiation potential of fibroblasts. (**E**) Bar plots showing enriched GO terms and differentially regulated pathways identified through analysis of highly variable markers in Fibro-c1. (**F**,**G**) Pseudotime trajectory of fibroblasts in a two-dimensional state-space predicted by Monocle 2. Each dot represents a single cell, colored according to its cluster label. (**H**) Heatmap depicting the activity of variable transcription factors across all fibroblast subclusters.

Analysis of cell populations affected by colitis revealed a global decrease in fibroblasts, suggesting that one or more specific subsets of these intestinal stromal cells are sensitive to inflammatory cues. To demonstrate intestinal fibroblast subpopulation dynamics during colitis and recovery, we isolated 7572 fibroblasts and assigned them to seven clusters (Figure 2A). All clusters expressed established fibroblast markers like *Col1a1* and *Col3a1* [21] (Figure 2B). Fibro-c0 and c2 displayed high expression of *Has1* and *Pi16*, indicating pan-tissue extracellular matrix production [22,23]. Fibro-c3 and c4 exhibited elevated *Adamdec1*, a marker of activated fibroblasts, with c4 uniquely expressing the inflammatory cell chemokine *Ccl8*. Fibro-c6 showed high expression of *Acta2* and *Tagln*, characteristic of myofibroblasts. Notably, Fibro-c1 expressed high levels of *Sfrp2*, a stem/mesenchymal cell marker, and *Nrg1*, encoding neuregulin-1 as an epidermal growth factor family ligand for epithelial cell repair [24,25]. Fibro-c1 was scarce in the absence of inflammation (‘None’ stage, Figure 2C). However, these cells enriched during the early and middle stages of repair in inflamed tissue, suggesting a role in early epithelium healing (Figure 2C). Cytotrace analysis confirmed the stemness potential of Fibro-c1 compared to other differentiated clusters (Figure 2D). Gene Ontology (GO) analysis revealed Fibro-c1 involvement in organ morphogenesis, mesenchymal cell differentiation, and wound-healing regulation (Figure 2E). Pseudotemporal analysis suggested Fibro-c1 functions as the starting point, differentiating into three lineages (Figure 2F). One pathway leads to Fibro-c0 and c2, specializing in extracellular matrix production (Figure 2G). Fibro-c3 and c6 represent the endpoints of separate lineages, and Fibro-c4 and c5 appear as transient intermediate states (Figure 2G). These findings suggest that Fibro-c1 is a stem-like fibroblast crucial for mucosal healing after acute inflammation-induced epithelial damage. DoRothEA analysis (Figure 2H) revealed activation of transcription factors in Fibro-c1, including *Trp53*, *Myc*, *Hif1a,* and *Nrf1*, associated with cell proliferation and metabolism [26,27,28]. Notably, Tead family members *Tead1–4*, known to stimulate Nrg1 secretion [29,30], were also activated. Additionally, activation of Stat1 and Stat3, linked to inflammatory responses [31,32], suggests adaptation of Fibro-c1 to inflammation.

### 2.3. Dynamics of Myeloid and Epithelial Cells during the Colitis and Recovery Phases

**Figure 3:**
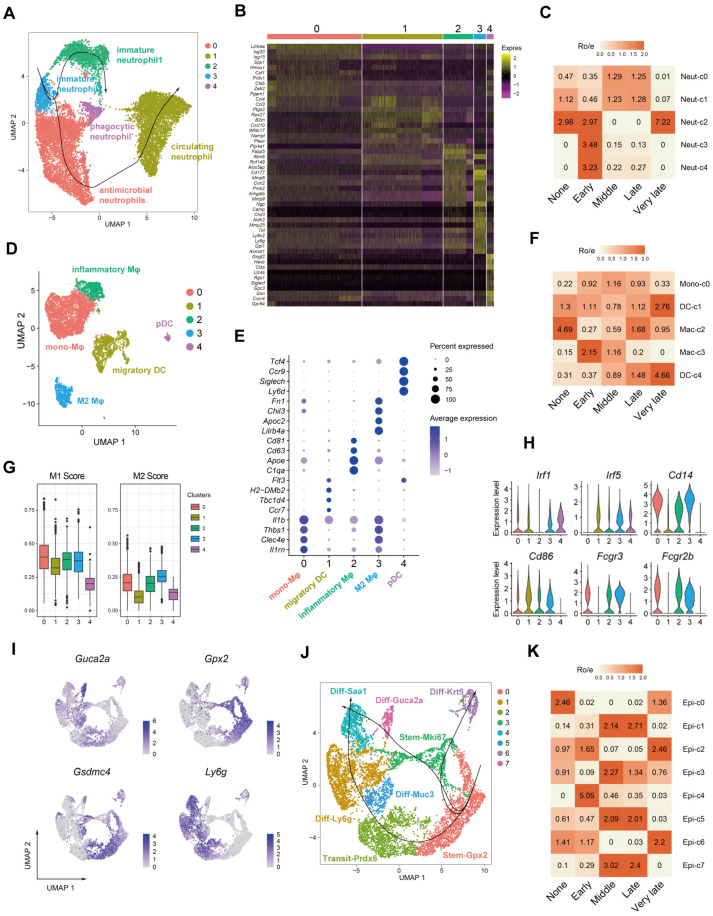
Identification and characterization of infiltrated inflammatory myeloid and epithelial cells during colitis-caused epithelial healing. (**A**) UMAP projection and pseudotime trajectories of neutrophils colored by cluster. (**B**) Heatmap of the top 10 most variable markers for the 5 neutrophil clusters. (**C**) Estimated tissue prevalence of each neutrophil subcluster using the Ro/e score. (**D**) UMAP projection of other myeloid cells except neutrophils colored by cluster. (**E**) Bubble plot depicting expression levels of canonical marker genes in each myeloid cell cluster. (**F**) Estimated tissue prevalence of each myeloid cell cluster using the Ro/e score. (**G**) Boxplot depicting M1 or M2 scores of dendritic cells (DCs) and macrophages. The median is represented by the middle line of the boxplot, with hinges indicating the interquartile range (IQR). (**H**) Violin plots showing expression levels of selected M1 signature genes across macrophage subclusters. (**I**) Estimated tissue prevalence of each epithelial subcluster using the Ro/e score. (**J**) UMAP projection and pseudotime trajectories of epithelial cells colored by cluster. (**K**) UMAP plots of marker genes referenced for epithelial cells during tissue healing in colitis.

We then investigated the infiltrating immune cell populations in the colonic mucosa using the same scRNA-seq data. Neutrophils were categorized into five distinct clusters (Neut-c) based on their gene expression profiles (Figure 3A,B). Neut-c2 and c3, expressing markers like *Ngp*, *Camp*, *Chil3*, *Mmp8*, and *Mmp9*, represented immature neutrophils [33] (Figure 3B). These immature populations were enriched during early healing stages, coinciding with ongoing inflammation (Figure 3C). Neut-c4, also prevalent in the early colitis phase, expressed genes associated with phagocytosis and reactive oxygen species (ROS) generation (*Gngt2*, *Ltc4s*, *Gpr84*) [34] (Figure 3B). In contrast, Neut-c0, dominant during later stages, expressed interferon-stimulated genes (*Isg20*, *Isg15*) (Figure 3B). This suggests a potential role in bacterial defense via interferon pathways, possibly due to increased bacterial exposure following epithelial damage. Neut-c1 expressed genes indicative of recently recruited neutrophils from the bloodstream (*Wfdc17*, *Slpi*, *Wfdc21*) (Figure 3B). By Slingshot analysis, we identified the differential tract of neutrophils in the context of colitis and found that immature intestinal neutrophils developed into phagocytic terminal and antimicrobial and further circulating terminal subsets, respectively (Figure 3A). We also identified two distinct dendritic cell (DC) populations in the previously identified ‘Macrophage’ cluster from Figure 1. DC-c1 expressed markers of migratory DCs (*Ccr7*, *Tbc1d4*, *H2-DMb2*) [35,36], while DC-c4 expressed markers of plasmacytoid DCs (*Siglech*, *Ly6d*) [37] (Figure 3E). Meanwhile, three macrophage clusters emerged following further analysis (Figure 3D–F), and M1 and M2 scores were utilized to assess their functional phenotypes during colitis-induced mucosal healing. DCs exhibited the lowest M1 and M2 scores, while mono- and inflammatory macrophages displayed higher M1 scores [38] (Figure 3G), indicating the dominance of pro-inflammatory macrophages during colitis (related to *Il1b*, *Irf1*, *Cd14*, *Fcgr3* expression level, Figure 3H). Mac-c3 had the highest M2 score (*Fn1*, *Chil3*), suggesting anti-inflammatory activity (Figure 3G,H). Epithelial cell analysis revealed *Gpx2* expression in Epi-c0 and c3 (Figure 3I,J), suggesting resident stem cells in healthy tissue. Epi-c0 and part of c2 represent progenitor cells located higher in the crypts (*Gsdmc4*^+^, Figure 3I,J). These pre-existing progenitors may contribute to repair in the middle and late stages (Figure 3K). Early healing is dominated by non-secretory epithelial cells [39,40] (*Ly6g*^+^ in Ep-c1, Figure 3I–K). As the estimated founder progenitors, *Gpx2*^+^ and *Mki67*^+^ cells eventually differentiated into *Krt5*^+^ or *Saa1*^+^ epithelial terminal subsets (Figure 3K), probably driving epithelial regeneration consequently.

### 2.4. IL-1 May Influence Nrg1–Erbb3 Interaction between Fibroblasts and Epithelial Cells

**Figure 4:**
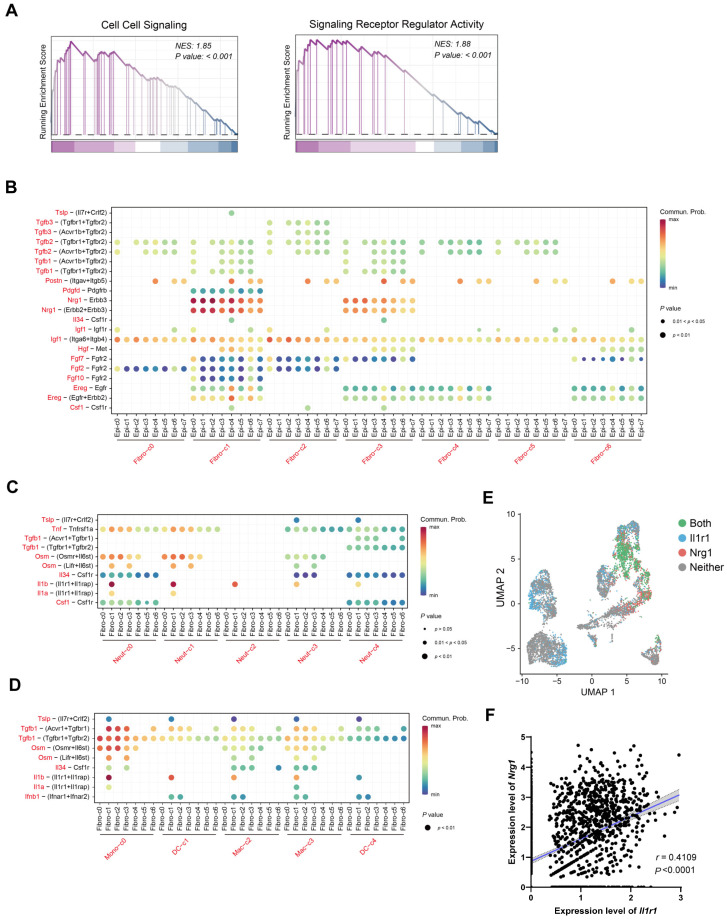
**Cell–cell interactions between stroma and inflammatory myeloid cells in epithelial healing.** (**A**) GSEA plots depicting significantly enriched pathways in Fibro-c1. NES indicates normalized enrichment score. (**B**–**D**) Bubble plot showing significant interactions between fibroblasts and other epithelial cell clusters mediated by ligand–receptor pairs of cytokines and growth factors (**B**). Additional bubble plots depict ligand–receptor pairs of cytokines between neutrophils (**C**) or macrophages (**D**) and other fibroblast clusters. (**E**) UMAP plot showing the binary expression pattern of *Il1r1* and *Nrg1* in fibroblasts. Colors indicate cells expressing only *Il1r1* (blue), only *Nrg1* (pink), both genes (green), or neither (gray). (**F**) Correlation curves between *Nrg1* and *Il1r1* expression in Fibro-c1, with each dot representing a single cell (n = 1499). *p* value is derived from the Spearman correlation test.

Gene Set-Enrichment Analysis (GSEA) revealed significant enrichment of cell–cell signaling and signaling receptor regulator activity pathways in Fibro-c1 stem-like fibroblasts (Figure 4A). This suggests a potential role for stem-like fibroblasts in intercellular communication during colonic epithelial healing. CellChat analysis identified the Nrg1–Erbb3 signaling pathway as a key mediator of interaction between stem-like fibroblasts and epithelial cells, particularly the *Gpx2*^+^ progenitor and *Ly6g*^hi^ epithelium (Figure 4B, Supplementary Figure S1A). Nrg1 secreted by these ‘stem’ fibroblasts likely acts as a growth factor facilitating epithelial repair. Given the activation of inflammatory transcription factors in Fibro-c1 stem-like fibroblasts (Figure 2H), we further investigated their communication with infiltrating immune cells. Fibro-c1 expressed *Il1r1*, a receptor for the pro-inflammatory cytokine IL-1β secreted by neutrophils and macrophages (Figure 4C,D, Supplementary Figure S1B,C). Notably, *Il1r1* expression in fibroblasts was restricted to Fibro-c1 and co-localized with *Nrg1* (Figure 4E). Furthermore, a positive correlation was observed between *Il1r1* and *Nrg1* expression within Fibro-c1 (Figure 4F). These findings suggest that neuregulin-1 by stem-like fibroblasts, crucial for intestinal epithelial repair, might be modulated by IL-1 signaling from inflammatory myeloid cells.

### 2.5. Myeloid Cell Accumulation and IL-1R and Nrg1 Upregulation in the Process of Colitis-Induced Epithelial Healing

**Figure 5:**
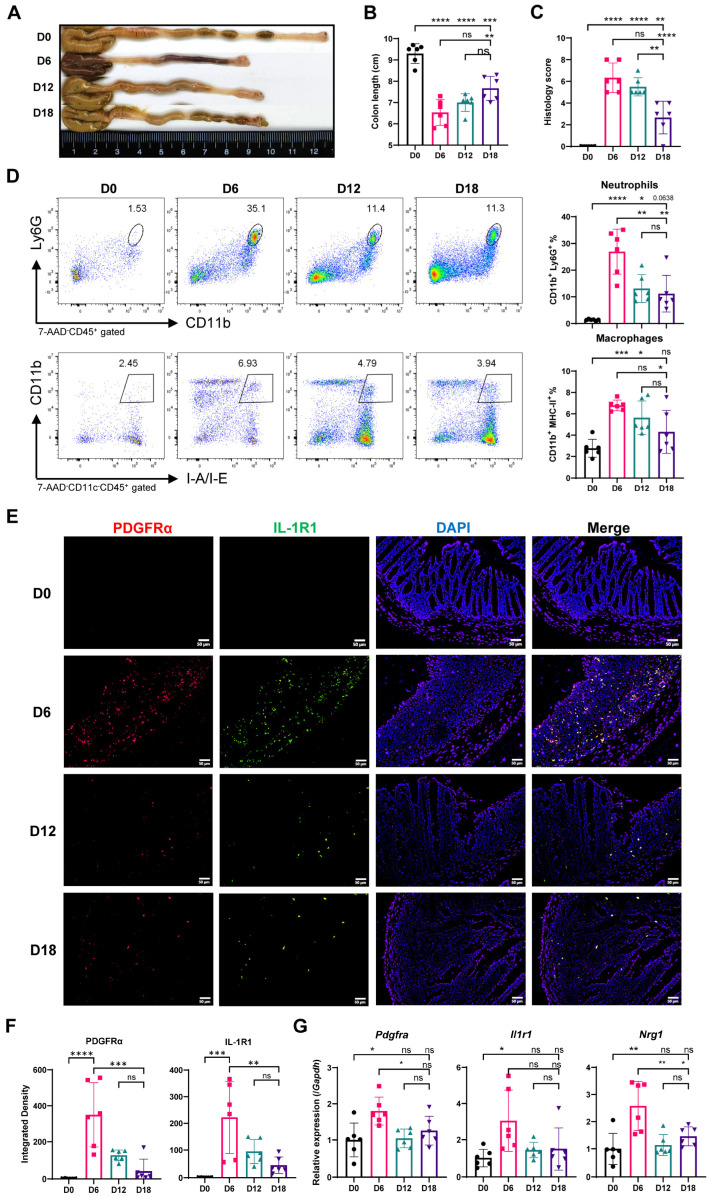
**Neutrophils and macrophages exhibit dynamic changes during DSS-induced colitis.** (**A**–**G**): Mice were treated with DSS in drinking water for 6 days (D6) followed by recovery with normal water. D18 and D12 represent recovery periods of 12 and 6 days after DSS treatment, respectively. D0 represents control mice with no DSS exposure (n = 6/group for all). (**A**) Representative images of colons from each group. (**B**) Colon length was measured after euthanasia. (**C**) Histological score of colon tissue sections. (**D**) Flow cytometry analysis of neutrophils (CD11b^+^ Ly6G^+^) and macrophages (CD11b^−^ Ly6G^+^). (**E**) Immunofluorescence staining of PDGFRα (red), IL-1R1 (green), and DAPI (blue) in colon tissues (n = 6/group). The scale bar represents 50 μm. (**F**) Integrated fluorescence density of PDGFRα and IL-1R1 (n = 6/group). (**G**) Relative mRNA expression of *Pdgfra*, *Il1r1*, and *Nrg1* in mouse colon tissues was measured by qPCR (n = 6/group). Data in (**A**–**G**) are representative of two independent experiments. Data in (**B**–**D**,**F**,**G**) are shown as the mean ± SD, followed by one-way ANOVA with Bonferroni’s multiple-comparisons test. (ns: not significant, * *p* < 0.05, ** *p* < 0.01, *** *p* < 0.001, **** *p* < 0.0001).

To characterize the pathological phenotype at each stage of epithelial healing, we established DSS-induced colitis in mice at representative time points (D0, D6, D12, D18). Body weight and disease activity index were monitored daily to assess disease severity (Supplementary Figure S2A,B). As expected, colon length was significantly reduced during early healing (D6) and progressively increased at later stages (D12, D18) (Figure 5A,B, Supplementary Figure S2C). On D6, colons displayed the most severe infiltration by inflammatory cells and substantial disruption of tissue architecture (Figure 5C, Supplementary Figure S2D). With extended healing (D12, D18), colonic inflammation subsided, and tissue architecture approached a pre-disease state. IL-1β production, primarily by neutrophils and macrophages, peaked at D6 and decreased throughout recovery (Supplementary Figure S1C). Flow cytometry analysis (Figure 5D) revealed a minimal baseline presence of neutrophils and macrophages (D0). During early healing (D6), both cell types increased significantly, with a more pronounced rise in neutrophils (1.72% to 35.3%) compared to macrophages (2.45% to 6.93%). By the middle (D12) and late (D18) stages, neutrophil and macrophage abundance declined but remained elevated compared to baseline (D0). These findings confirm the dynamic changes in neutrophil and macrophage populations during early repair observed through scRNA-seq analysis.

Interleukin-1 (IL-1) signaling plays an important role in tissue repair, with its receptor, IL-1R1, expressed by various cell types including colonic fibroblasts [8,41,42]. The IL-1 signaling pathway is thought to be crucial for colonic tissue regeneration after injury [17]. In our DSS-induced colitis model, the fibroblast marker PDGFRα displayed a distinct expression pattern, being undetectable at baseline (D0), peaking during early repair (D6), and decreasing by D12 and D18 (Figure 5E–G). This suggests a prominent role for fibroblasts specifically in early healing. Notably, IL-1R1 expression mirrored PDGFRα, with substantial co-localization, suggesting co-expression of these markers (Figure 5E–G). As the promoter of epithelial repair, Nrg1 [16] peaked during early healing and returned to baseline levels by the middle and late stages (Figure 5G).

### 2.6. Most Neuregulin-1-Secreting Fibroblasts Express IL-1R1 and IL-1 Signaling Suppresses Nrg1 Expression under Inflammation Condition

**Figure 6:**
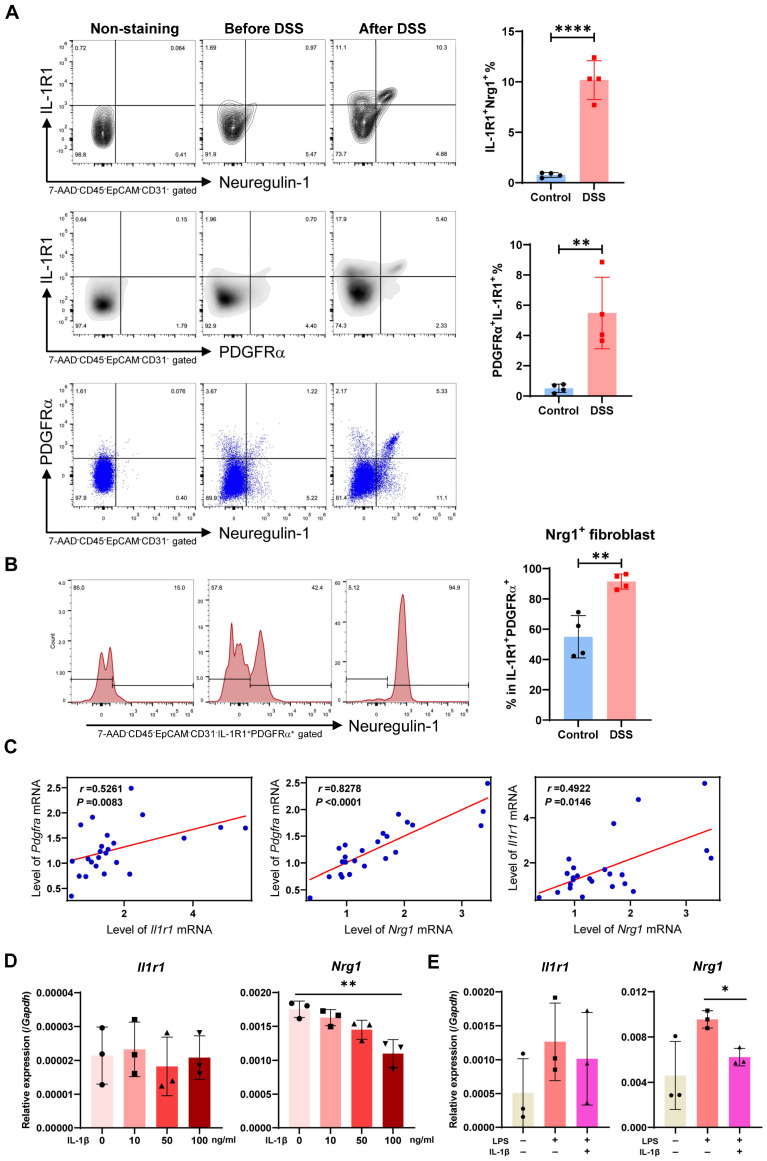
**Neuregulin-1 is co-expressed with IL-1R1 in fibroblasts and is suppressed by IL-1 signaling.** (**A**,**B**) Mice were treated with 3% DSS for six days, and colonic cells were harvested to examine the proportions of neuregulin-1^+^IL-R1^+^, PDGFRα^+^IL-1R1^+,^ and neuregulin-1^+^ PDGFRα^+^ population in whole colonic fibroblast (**A**) and neuregulin-1^+^ population in PDGFRα^+^ IL-1R1^+^ fibroblast (**B**) by flow cytometry (n = 4/group). (**C**) Correlation analysis between normalized expression levels of *Pdgfra*, *Il1r1*, and *Nrg1* in mouse colon tissues (n = 18). Spearman’s correlation coefficient (r) is shown. (**D**) Mice were treated with 3% DSS for six days, and colonic CD45-EpCAM-CD31-fibroblasts were sorted and treated with recombinant mouse IL-1β for 24 h. Relative mRNA expression of *Il1r1* and *Nrg1* in these fibroblasts was measured by qPCR (n = 3/group). (**E**) Lung fibroblasts were obtained from normal C57BL/6J mice and were stimulated with LPS for 24 h followed by IL-1β addition for another 24 h. Relative mRNA expression of *Il1r1* and *Nrg1* in lung fibroblasts was measured by qPCR (n = 3 replicates/group). Data in (**A**–**E**) are representative of two independent experiments. Data in (**A**,**B**,**D**,**E**) are shown as the mean ± SD, followed by one-way ANOVA with Bonferroni’s multiple-comparisons test. (* *p* < 0.05, ** *p* < 0.01, **** *p* < 0.0001).

After treating mice with DSS for 6 days, we detected that both neuregulin-1 and IL-1R1 protein levels were upregulated in colonic fibroblasts compared to steady-state controls (Figure 6A). Notably, a majority of these neuregulin-1-producing fibroblasts also co-expressed IL-1R1. Furthermore, over 90% of the IL-1R1^+^PDGFRα^+^ fibroblast population secreted neuregulin-1 during the early phase of epithelial repair, compared to only 50% in the normal state (Figure 6B), suggesting an enhanced capacity of intestinal fibroblasts to repair epithelial injury, potentially regulated by pro-inflammatory signaling. Additionally, a strong positive correlation was observed between mRNA expression of PDGFRα, Nrg1, and IL-1R1 in mouse colon tissues (Figure 6C). By analyzing another scRNA-seq public dataset from DSS-treated mouse colonic cells (GSE168033), we confirmed a positive correlation between *Pdgfra*, *Nrg1,* and *Il1r1* expression in colonic fibroblasts (Supplementary Figure S3A–C), suggesting their coordinated contribution to the epithelial healing process.

To clarify the direct influence of IL-1 signaling on neuregulin-1 expression in fibroblasts, we employed two in vitro models. The first model utilized mice with acute colitis induced by 3% DSS for six days. CD45-EpCAM-CD31-fibroblasts were sorted from colonic tissue and treated with recombinant mouse IL-1β for 24 h. *Nrg1* mRNA expression in these fibroblasts displayed a dose-dependent decrease following IL-1β stimulation while *Il1r1* expression remained unaffected (Figure 6D). This finding was further corroborated in a second model using fibroblasts from a distinct organ—lung fibroblasts. These cells were stimulated with LPS for 24 h to mimic pre-existing inflammation, followed by IL-1β treatment for another 24 h. Similar to the first model, Nrg1 expression was significantly downregulated by IL-1β without altering *Il1r1* expression (Figure 6E). Integrating these findings with the observation of IL-1β expression by intestinal neutrophils and macrophages from scRNA-seq data, we propose that inflammatory myeloid cells directly suppress Nrg1 expression in fibroblasts through IL-1 signaling. This mechanism may contribute to impaired intestinal epithelial recovery after colitis.

### 2.7. Co-Expression of NRG1 and IL1R1 Is also Observed in Intestinal Fibroblasts of IBD Patients

**Figure 7:**
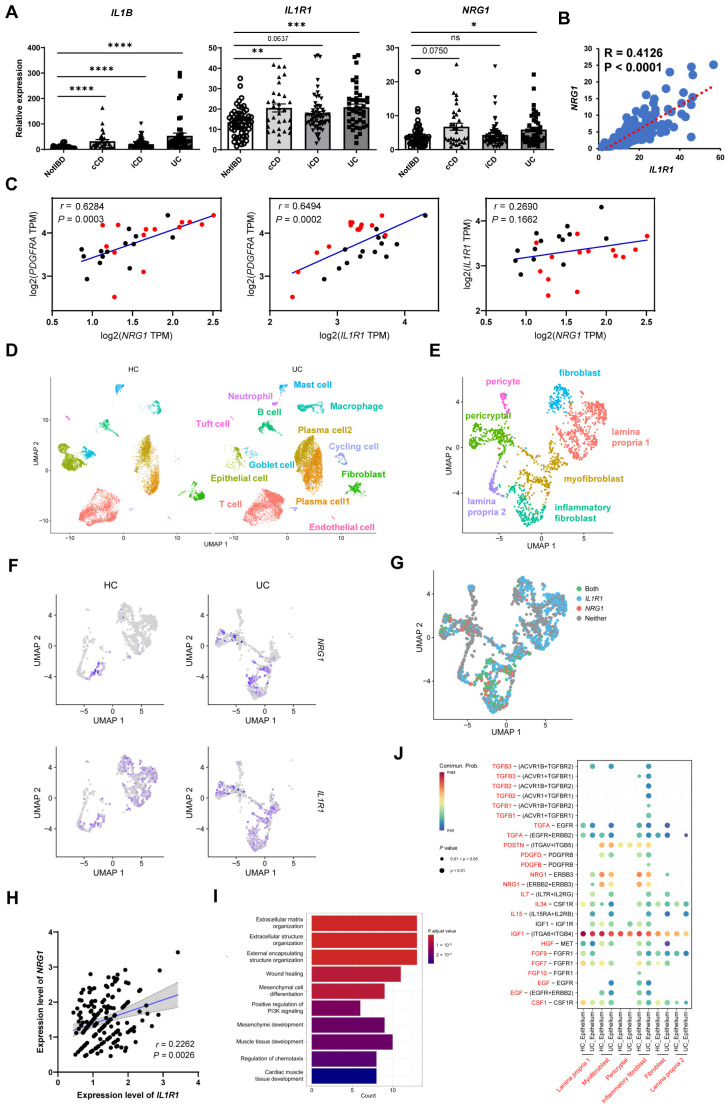
**NRG1-IL-1R1 co-expressing intestinal fibroblasts also exist in IBD patients** (**A**) Transcriptional levels of *IL1B*, *IL1R1*, and *NRG1* genes expressed in colon tissues from non-IBD control, patients with colonic CD, ileal CD, and UC diseases were determined by analyzing a public bulk RNA-seq dataset (GSE117993, Not IBD n = 55, cCD n = 31, iCD n = 60, UC n = 43). Data are shown as the mean ± SD, followed by the Kruskal–Wallis test with Dunn’s multiple-comparisons test. (**B**) Correlation analysis between expression levels of *IL1R1* and *NRG1* in colon tissues of all individuals from GSE117993 (n = 189). Spearman’s correlation coefficient (r) is shown. (**C**) Correlation analysis between expression levels of *PDGFRA*, *IL1R1*, and *NRG1* in colon tissues of IBD patients from the GSE128682 dataset (n = 44). Pearson’s correlation coefficient (r) is shown. (**D**) UMAP projection of 27,456 single cells from human healthy and UC cohorts, split by disease state and colored by cluster. HC, health control; UC, ulcerative colitis. (**E**) UMAP projection of 1643 fibroblasts, colored by cluster. (**F**) Feature plot showing the expression of *NRG1* and *IL1R1*, split by disease state. (**G**) UMAP projection depicting the binary expression pattern of *IL1R1* and *NRG1* in the identified fibroblast population, with colors indicating cells expressing only *IL1R1* (blue), only *NRG1* (pink), both genes (green), or neither (gray). (**H**) Correlation curves between *NRG1* and *IL1R1* in *NRG1*^+^*IL1R1*^+^ fibroblasts, with each dot representing one cell (n = 175). *p* value from Spearman correlation test. (**I**) Bar plots showing the enriched items and differential pathways of GO analysis based on highly variable markers of *NRG1*^+^*IL1R1*^+^ fibroblasts. (**J**) Bubble plot showing significant interactions between fibroblasts and epithelial cell clusters by ligand–receptor pairs of cytokines and growth factors. (* *p* < 0.05, ** *p* < 0.01, *** *p* < 0.001, **** *p* < 0.0001).

To demonstrate whether NRG1 and IL-1R were also co-expressed on colonic fibroblasts in IBD patients, we first analyzed their expression levels in the whole inflamed colon tissues from patients with UC and Crohn’s disease (CD) (GSE117993). Expression levels of *IL1B*, *IL1R1*, and *NRG1* were all upregulated in these IBD patients compared with healthy control (Figure 7A), and *IL1R1* and *NRG1* expression exhibited a strong positive correlation (Figure 7B). This correlation was also observed in UC patients, both active and in remission (GSE128682), mirroring the findings in our mouse model (Figure 7C). By analyzing scRNA-seq data of UC patients from a public GEO dataset (GSE214695), we assigned colonic cells for 13 clusters (Figure 7D, Supplementary Figure S3D), and isolated and identified 7 clusters of stromal cells (Figure 7E, Supplementary Figure S3E). Compared to healthy controls, UC patients displayed upregulated *NRG1* expression specifically in inflammatory fibroblasts and myofibroblasts (Figure 7F). Furthermore, co-localization of *NRG1* and *IL1R1* within the same fibroblast clusters was observed (Figure 7G). A positive correlation of *NRG1* and *IL1R1* in *NRG1*^+^*IL1R1*^+^ fibroblasts was also exhibited (Figure 7H), and these double-positive cells also displayed functional characteristics associated with extracellular matrix organization and wound healing (Figure 7I), consistent with our mouse data. Additionally, NRG1–ERBB2/3 interaction between both inflammatory fibroblasts and myofibroblasts with epithelial cells was observed in both UC and healthy control samples (Figure 7J). These results suggest that NRG1-mediated epithelial repair may also regulated by IL-1 signaling in human intestinal fibroblasts.

## 3. Discussion

Following epithelial cell necrosis and detachment in ulcerative colitis, restoring the intestinal mucosa is a complex process exceeding the established role of intestinal stem cell proliferation and differentiation [43,44]. The tissue microenvironment plays a crucial role, highlighting the importance of interactions between diverse cellular constituents. Notably, fibroblasts, abundant stromal cells, emerge as key players in orchestrating tissue repair [45].

Our study identified a unique subpopulation of fibroblasts enriched during early colitis healing, characterized by the expression of neuregulin-1. This mesenchymal-derived Nrg1 likely acts as a potent facilitator of intestinal stem cell (ISC) regeneration after injury. The restorative function of Nrg1 in epithelial repair, mediated through its binding to ErbB receptors on epithelial cells, particularly via the Nrg1–Erbb3 ligand–receptor pathway, is well-supported by bioinformatics analyses, in vitro, and in vivo studies [15,16].

The significant activation of IL-1 receptor signaling on these wound-healing fibroblasts suggests a critical role in UC. Infiltration of inflammatory cells, particularly neutrophils and macrophages, is the primary source of IL-1β, which activates these fibroblasts. This establishes a cellular communication network within the microenvironment, potentially influencing fibroblast-mediated epithelial repair. Previous studies have documented interactions between inflammatory cells and fibroblasts in UC. For example, IL-1β-activated FAP^+^ fibroblasts recruit neutrophils through chemokines like CXCL5, CXCL6, and CXCL8 [46]. In the present study, we found that IL-1 signaling suppressed the expression of Nrg1 in intestinal fibroblasts, indicating a distinct role for IL-1β signaling in suppressing early epithelial repair caused by inflammation.

Our findings implied that neutrophils and macrophages acted as an upstream of IL-1R-expressing Fibro-c1 fibroblasts, facilitating Stat1 and Stat3 activation through Il-1β signaling, which was upregulated in these fibroblasts. Notably, IL-1R1 expression is not limited to fibroblasts in colitis; it is also observed in mesenchymal cells during autoimmune diseases, intestinal inflammation, and solid tumors, where it exhibits diverse functions [17,47,48]. Although IL-1 signaling was previously reported as a promoter of lung regeneration as demonstrated in alveolar organoids and lung tissue [42], its suppressive role in intestinal epithelial healing in UC found in this study indicates its original function to facilitate inflammation, suggesting a context-dependent effect of this cytokine.

The high-resolution advantage of scRNA-seq allowed us to depict the dynamic landscape of various cells. By analyzing the public transcriptomic data of different time points throughout the progression of colitis, we aimed to identify therapeutic targets that could aid in repairing damaged mucosa and enhancing the quality of life for patients with colitis. Single-cell RNA-seq analysis in the present study revealed significant heterogeneity among colonic fibroblasts. *Grem1* and *Rspo3*, markers associated with a distinct fibroblast population promoting intestinal stem cell (ISC) proliferation, were not co-expressed with *Il1r1* in Fibro-c1 stem-like fibroblasts. This finding highlights the diverse functional repertoire of fibroblasts in DSS-induced colitis. However, activation of Tead transcription factors within stem-like fibroblasts suggests robust Nrg1 secretion, potentially explaining their role in epithelial repair. Immunofluorescence staining and flow cytometry further supported this notion, revealing a transient co-expression of IL-1R1, neuregulin-1, and PDGFRα during the early healing phase. These neuregulin-1^+^IL-1R1^+^ fibroblasts were scarce in non-inflamed tissues, suggesting their specific involvement in epithelial healing. Additionally, bioinformatics analysis of this fibroblast subset identified expression of the stemness-related gene *Sfrp2*, indicating their potential to differentiate during the early stages of mucosal repair.

Our research still has certain limitations. *Nrg1*^+^*Il1r1*^+^ fibroblasts were absent in non-inflamed colons, suggesting that some inflammation-associated driving factor(s) promoted their enrichment and proliferation in the early stage and induced the expression of *Nrg1* and *Il1r1*. These driving factors need further identification. After reaching the peak of inflammation, these Nrg1^+^ fibroblasts were subjected to IL-1β. It remains unclear whether they gradually differentiated into other fibroblasts or underwent apoptosis, which needs further exploration.

The effect of intervening with these stem-like Nrg1^+^ fibroblasts to improve the prognosis of colitis patients is still far from being achieved. Neutralization of IL-1β or inhibition of its receptor may be constrained due to their extensive impacts on various tissues. Although the role of NRG1 in promoting intestinal repair has been confirmed in vivo, no clinical trials of recombinant human NRG1 are reported for the treatment of IBD. Future attempts might be important to assess the efficacy of both NRG1 enhancement and IL-1-IL-1R signaling blockade in the clinical management of colitis.

Our study employed bioinformatics analysis to identify a unique population of colonic fibroblasts co-expressing Nrg1 and IL-1R1. These *Nrg1*^+^*Il1r1*^+^ fibroblasts exhibit differentiation potential and actively contribute to epithelial repair during the early stages of ulcerative colitis recovery. This finding underscores the remarkable heterogeneity of the fibroblast population in colitis. Furthermore, the intricate cellular network linking infiltrating inflammatory cells, fibroblasts, and the intestinal epithelium highlights an important role for IL-1 signaling in the inflamed gut to suppress epithelial regeneration through fibroblast-mediated mechanisms.

## 4. Materials and Methods

### 4.1. Data Collection, Processing, and Cluster Analysis

Single-cell RNA-sequencing (scRNA-seq) data of mice (GSE193342 and GSE168033) and patients (GSE214695) were obtained from the Gene Expression Omnibus (GEO) database. Colitis in mice was induced by administering dextran sulfate sodium (DSS) in drinking water for six days, followed by regular water. Mucosal samples for sequencing were collected from mice at baseline (D0, pre-DSS), early (D6, post-DSS), middle (D12), late (D18), and very late (D42) stages of epithelium healing after DSS administration. The downloaded count matrix was processed using the Seurat package (v4.1.1) within the R programming environment (v4.1.3) to create a Seurat object [49]. The object underwent normalization and doublet removal with DoubletFinder (v2.0.3) [50]. Low-quality cells expressing mitochondrial genes exceeding 25% or with unique gene counts outside the range of 200–5000 were excluded. The FindVariableFeatures() function identified the top 2000 highly variable genes for dimensionality reduction. UMAP analysis was performed using the top 20 significant principal components (PCs) determined by the ElbowPlot() function. The FindNeighbors() and FindClusters() functions were applied for cell clustering. Cell identity was assigned based on the expression of established marker genes. Each major cell type was identified at a low resolution, while the finer subclusters were distinguished using high-resolution clustering. The analysis carried out in Figure 1, Figure 2, Figure 3 and Figure 4 by using the same public single-cell sequencing datasets. Additionally, bulk RNA-Seq data from IBD patients were retrieved from GSE117993 and GSE128682 [51].

### 4.2. Tissue Prevalence of Clusters

To assess the spatial distribution of each cell population across tissues, we calculated the observed-to-expected (O/E) ratio for each cluster in different tissue environments [52]. The chi-square test was used to determine the expected cell abundance for each combination of cell cluster and tissue type. An O/E ratio greater than 1 indicates enrichment of a specific cell type in a particular tissue compared to random distribution. Conversely, an O/E ratio below 1 suggests depletion of that cell type relative to random expectation in that tissue.

### 4.3. GO Enrichment and GSEA Analysis

Cluster-specific markers were identified using the FindAllMarkers() function in Seurat with the following parameters: only.pos = TRUE and min.pct = 0.25. Wilcoxon rank-sum tests were employed to assess the statistical significance of these markers between clusters. For each cluster, the top 100 unique markers were subjected to Gene Ontology (GO) and Gene Set-Enrichment Analysis (GSEA) using the clusterProfiler package (v4.2.2) [50]. Significantly enriched terms and pathways were identified with a *p*-value cutoff of 0.05 to ensure the capture of relevant biological insights.

### 4.4. CytoTRACE Analysis

We employed CytoTRACE (version 0.3.3) [53], an unsupervised method for inferring differentiation trajectories from single-cell RNA-seq data. The analysis leveraged established pathways within CytoTRACE to complement trajectory inference. The metadata and count matrix of the cell population to be analyzed served as input data for the Cytotrace() function pipeline. Visualization of the inferred trajectories was achieved using the plotCytoTRACE() function.

### 4.5. Pseudotime Trajectory Analysis

Cellular pseudotime trajectories were inferred using Monocle2 (version 2.26.0) and Slingshot (version 2.10.0) [54] to explore transitions between cell states. The Seurat object was reorganized into a CellDataSet object through the newCellDataSet() function. Ordering genes were selected based on mean expression (>0.1) and variability, following Monocle2 guidelines. Dimensionality reduction was achieved using the default parameters of the DDRTree() function. Leveraging the identification of stem-like fibroblasts, these cells were designated as the initial state for trajectory inference using Monocle2’s “order” function. Further refinement was achieved by splitting based on cluster identity. For Slingshot analysis, the Seurat object of cell type to be analyzed was transformed into a SingleCellExperiment object. The coordinate matrix of cells in UMAP dimensionality reduction and the label vector of subclusters were used to the run slingshot() function. Immature Neut-c3 and epithelial progenitor cells Epi-c0 were selected as possible starting points.

### 4.6. Dorothea Analysis

Dorothea (version 1.6.0) was employed to extract mouse-relevant regulons, categorized by interaction confidence levels (A,B,C) [55]. Viper scores were subsequently calculated for these regulons and compiled into a data frame through the run_viper() function. This facilitated a comprehensive evaluation of inter-population variation in Viper scores, enabling the selection of differentially expressed transcription factors (TFs) for visualization across all identified cell clusters.

### 4.7. Cell–Cell Interaction Analysis

Cell–cell interactions were investigated using CellChat (version 1.5.0) [56]. This software infers cellular communication based on the expression of known ligand–receptor pairs from CellChatDB [56]. CellChatDB.mouse and CellChatDB.human were, respectively, utilized for the analysis of corresponding species in this study. An object containing annotated cell types was created in CellChat. Focusing on “Secreted Signaling” pathways within CellChatDB.mouse, ligand–receptor pairs were employed to quantify interaction frequencies with computeCommunProb() and computeCommunProbPathway() functions (raw. use set to FALSE). The CellChat object was then integrated using aggregateNet() to provide a comprehensive overview of intercellular communication dynamics. The interaction between expected cell types were achieved by adjusting the sources.use and targets.use parameters in the netVisual_bubble() function.

### 4.8. DSS-Induced Colitis

C57BL/6J male mice aged 7–10 weeks were purchased from the Guangdong Medical Laboratory Animal Center and housed in environmentally controlled specific pathogen-free (SPF) conditions at the animal facility of Zhongshan School of Medicine, Sun Yat-sen University. Experimental colitis was induced by administering 1.5% dextran sulfate sodium (DSS, 36–50 kDa; MP Biomedicals, Illkirch, France) in drinking water. Mice were assigned to groups representing different stages of recovery: early (D6), middle (D12), or late (D18). The DSS treatment regimen for each group involved administration for 6 days followed by a switch to normal drinking water for D6 (D12 group), D12 (D18 group), or no recovery period (D6 group). D0 mice received normal drinking water throughout. Colitis severity was monitored daily using a previously described disease activity index (DAI) scoring system [57] encompassing diarrhea, rectal bleeding, and weight loss. Diarrhea: 0, normal; 2, loose stools; 4, watery diarrhea. Blood in stool: 0, normal; 2, slight bleeding; 4, gross bleeding. Weight loss: 0, none; 1, 1% to 5%; 2, 5% to 10%; 3, 10% to 15%; 4, >15%. The DAI was calculated as the average of these individual scores. Mice were sacrificed at D6, D12, or D18 after the initial DSS treatment. All animal experiments adhered to institutional ethical regulations and guidelines as approved by the Experimental Animal Management and Use Committee of Sun Yat-sen University (approval number: 2021001262).

### 4.9. Cell Preparation

Colonic lamina propria (CLP) cell isolation was performed as previously described [58]. Briefly, colonic tissues were cut into 1 mm pieces and incubated in HBSS containing 3 mM EDTA for 2 × 20 min at 37 °C with shaking. Following washes with HBSS, the tissue pieces were digested for 120 min at 37 °C with shaking in a complete RPMI medium supplemented with 200 U/mL collagenase (Cat# C2139; Sigma-Aldrich, St. Louis, MO, USA) and 5 U/mL DNase I (Sigma-Aldrich). The cell suspension was then filtered through 70 μm strainers to remove debris. After centrifugation, the cell pellet was resuspended in ACK lysis buffer to remove red blood cells. Isolated CLP cells were used for subsequent flow cytometry experiments.

### 4.10. Purification and Stimulation of Colonic Fibroblasts

CLP cells were labeled with biotin-conjugated anti-mouse CD326 (G8.8, Cat# 118203, Biolegend, San Diego, CA, USA), CD31 (MEC13.3, Cat# 102503, Biolegend) and CD45 (30-F11, Cat# 13-0451-82, eBioscience, San Diego, CA, USA) for 30 min, followed by anti-biotin microbeads. CD326, CD31, and CD45-positive cells were negatively sorted by EasyEights™ EasySep™ Magnet (Cat# 18103, STEMCELL, Vancouver, BC, Canada), which were identified as fibroblasts. Colonic fibroblasts were cultured in complete RPMI-1640 medium at a density of 1 × 10^4^ cells per well in 96-well plates supplemented with 0, 10, 50, and 100 ng/mL IL-1β (Cat# 50101-MNAE, Sino Biological, Beijing, China). After 24 h of treatment, colonic fibroblasts were harvested for RNA extraction and further qPCR analysis.

### 4.11. Culture of Lung Primary Fibroblasts

Lung fibroblasts were isolated from C57BL/6J mice. Briefly, the lung was excised, rinsed in ice-cold PBS, and minced into approximately 1 mm^3^ pieces. Following two washes and centrifugation at 1800 rpm for 5 min at 4 °C, the lung tissue fragments were resuspended in DMEM supplemented with 15% FBS, 1% penicillin-streptomycin, and plated evenly in a 10 cm dish. After 5 days in culture, adherent cells were harvested and subjected to negative selection using magnetic-activated cell sorting (MACS). Cells were labeled with a cocktail of biotin-conjugated antibodies against CD326 (G8.8, Cat# 118203, Biolegend), CD31 (MEC13.3, Cat# 102503, Biolegend), and CD45 (30-F11, Cat# 13-0451-82, eBioscience, San Diego, CA, USA), followed by incubation with anti-biotin microbeads. Unlabeled cells were retained as the purified lung fibroblast population. These primary lung fibroblasts were cultured in 15% FBS DMEM at a density of 1 × 10⁴ cells per well in 96-well plates. To mimic pre-existing inflammation, cells were stimulated with 1 μg/mL LPS (Cat# L2630, Thermo Fisher Scientific) for 24 h, followed by treatment with 10 ng/mL IL-1β (Cat# 50101-MNAE, Sino Biological, Beijing, China) for an additional 24 h. Stimulated lung fibroblasts were then harvested for RNA extraction and subsequent qPCR analysis.

### 4.12. Flow Cytometry

Fluorescently conjugated antibodies specific for mouse I-A/I-E (M5/114.15.2 Cat# 107614), CD11b (M1/70, Cat# 101216), CD11c (N418, Cat# 117322), CD45 (30-F11, Cat# 103116, 103126), Ly6G (1A8, Cat# 127606) and Ly6C (HK1.4, Cat# 128007) were purchased from BioLegend (San Diego, CA, USA). Antibodies against mouse PDGFRα (Cat# bs-0231R-AF594), IL-1R1 (Cat# bs-20698R-FITC), and neuregulin-1 (Cat# bs-1817R-Cy5) were purchased from Bioss (Beijing, China). 7-Amino-actinomycin D (7-AAD) viability staining solution was obtained from Tonbo Biosciences (San Diego, CA, USA). Isolated colonic lamina propria (CLP) cells were washed twice with FACS buffer (phosphate-buffered saline containing 2% fetal calf serum) and pretreated with 2.4G2 to block non-specific Fc receptor binding. Cells were then incubated with the antibodies mentioned above (1:250 dilution in FACS buffer) for 30 min at room temperature. In some experiments, biotin-conjugated antibodies were applied to cells for 30 min followed by incubation of Streptavidin-BV421. Stained single-cell suspensions were analyzed using an Attune NxT flow cytometer (Thermo Fisher Scientific, Waltham, MA, USA), and data were analyzed with FlowJo v10 software.

### 4.13. Real-Time RT-PCR

Total RNA was isolated from colonic tissues using the Mammalian Total RNA Miniprep Kit (Cat# AG11706, Sigma-Aldrich, St. Louis, MO, USA). Reverse transcription of RNA to cDNA was performed with the Evo M-MLV RT Master Mix (Agbio, Hunan, China) according to the manufacturer’s instructions. Quantitative real-time PCR (qRT-PCR) was then conducted using the SYBR Green Premix Pro Taq HS qPCR Kit (Cat# AG11718, Agbio) on a QuantStudio5 platform (Thermo Fisher Scientific, Waltham, MA, USA). Primer sequences used for qRT-PCR are listed in Supplementary Table S1. Gene expression levels were normalized to *Gapdh*.

### 4.14. Histological Analysis

Colon tissues were dissected and fixed in 10% formalin. Subsequently, paraffin-embedded tissues were sectioned at 5 μm thickness and stained with hematoxylin and eosin (H&E). Histological evaluation was performed using a previously described scoring system [59] that assessed epithelial integrity (E) and immune cell infiltration (I). Histology was scored as follows: epithelium (E), 0 = normal morphology; 1 = loss of goblet cells; 2 = loss of goblet cells in large areas; 3 = loss of crypts; 4 = loss of crypts in large areas; and infiltration (I), 0 = no infiltrate; 1 = infiltrate around the crypt basis; 2 = infiltrate reaching the lamina (L) muscularis mucosae; 3 = extensive infiltration reaching the L muscularis mucosae and thickening of the mucosa with abundant oedema; 4 = infiltration of the L submucosa. A total histological score was obtained by summing the E and I scores.

### 4.15. Immunofluorescence Staining

Immunofluorescence staining was performed on frozen colon tissue sections. Briefly, sections were blocked with 5% bovine serum albumin (BSA) in PBS for 1 h at room temperature. Subsequently, sections were incubated with primary antibodies against PDGFRα (Bioss, Cat# bs-0231R-AF594) and IL-1R1 (Bioss, Cat# bs-20698R-FITC) diluted in 1% BSA in PBS overnight at 4 °C. DAPI was used for nuclear counterstaining. Images were captured using an Olympus BX63 microscope (Olympus, Tokyo, Japan) and analyzed with ImageJ software (version 1.54h).

### 4.16. Statistical Analysis

For comparisons among three or more groups, one-way ANOVA with Bonferroni’s post hoc test or the Kruskal–Wallis test with Dunn’s multiple-comparisons test was used. The Pearson correlation coefficient was calculated using R software (version 4.1.3, R Foundation) or Prism v8.0 (GraphPad Software) to assess correlations. The Spearman correlation coefficient was calculated using Prism v8.0. All statistical analyses were performed with Prism v8.0. Statistical significance was set at *p* < 0.05.

## 5. Conclusions/Summary

- *Nrg1* is highly expressed in stem-like fibroblasts that appear early in the mouse colon after inducing colitis with DSS.
- Nrg1 signaling through Erbb3 might mediate communication between colonic fibroblasts and epithelial cells.
- During colitis, there are more myeloid cells releasing IL-1β, along with an increase in fibroblasts that express Nrg1 and IL-1 receptor 1.
- Stimulating mouse fibroblasts with IL-1β reduces Nrg1 expression.
- UC patients also show more *NRG1*^+^*IL1R1*^+^ colonic fibroblasts and NRG1–ERB interaction between fibroblasts and epithelial cells.

## Data Availability

The bulk RNA-seq and scRNA-seq data supporting the conclusion of this article are available in the Gene Expression Omnibus Repository (GSE193342, GSE168033, GSE214695, GSE117993 and GSE128682). The data from in vivo and in vitro experiments in this article are available by contacting the corresponding author.

## Supplementary Materials

The following supporting information can be downloaded at: https://www.mdpi.com/article/10.3390/ijms25084469/s1.

## References

[1] Rivière P., Li Wai Suen C., Chaparro M., De Cruz P., Spinelli A., Laharie D. (2024). Acute Severe Ulcerative Colitis Management: Unanswered Questions and Latest Insights. Lancet Gastroenterol. Hepatol..

[2] Le Berre C., Honap S., Peyrin-Biroulet L. (2023). Ulcerative Colitis. Lancet.

[3] Rudbaek J.J., Agrawal M., Torres J., Mehandru S., Colombel J.-F., Jess T. (2024). Deciphering the Different Phases of Preclinical Inflammatory Bowel Disease. Nat. Rev. Gastroenterol. Hepatol..

[4] Dignass A.U. (2001). Mechanisms and Modulation of Intestinal Epithelial Repair. Inflamm. Bowel Dis..

[5] Khor B., Gardet A., Xavier R.J. (2011). Genetics and Pathogenesis of Inflammatory Bowel Disease. Nature.

[6] Abraham C., Dulai P.S., Vermeire S., Sandborn W.J. (2017). Lessons Learned from Trials Targeting Cytokine Pathways in Patients with Inflammatory Bowel Diseases. Gastroenterology.

[7] Gordon I.O., Agrawal N., Goldblum J.R., Fiocchi C., Rieder F. (2014). Fibrosis in Ulcerative Colitis: Mechanisms, Features, and Consequences of a Neglected Problem. Inflamm. Bowel Dis..

[8] Song A., Zhu L., Gorantla G., Berdysz O., Amici S.A., Guerau-de-Arellano M., Madalena K.M., Lerch J.K., Liu X., Quan N. (2018). Salient Type 1 Interleukin 1 Receptor Expression in Peripheral Non-Immune Cells. Sci. Rep..

[9] Birchmeier C., Nave K.-A. (2008). Neuregulin-1, a Key Axonal Signal That Drives Schwann Cell Growth and Differentiation. Glia.

[10] Britsch S. (2007). The Neuregulin-I/ErbB Signaling System in Development and Disease. Adv. Anat. Embryol. Cell Biol..

[11] Mei L., Xiong W.-C. (2008). Neuregulin 1 in Neural Development, Synaptic Plasticity and Schizophrenia. Nat. Rev. Neurosci..

[12] Farkas J.E., Freitas P.D., Bryant D.M., Whited J.L., Monaghan J.R. (2016). Neuregulin-1 Signaling Is Essential for Nerve-Dependent Axolotl Limb Regeneration. Development.

[13] Bartus K., Galino J., James N.D., Hernandez-Miranda L.R., Dawes J.M., Fricker F.R., Garratt A.N., McMahon S.B., Ramer M.S., Birchmeier C. (2016). Neuregulin-1 Controls an Endogenous Repair Mechanism after Spinal Cord Injury. Brain.

[14] Rupert C.E., Coulombe K.L. (2015). The Roles of Neuregulin-1 in Cardiac Development, Homeostasis, and Disease. Biomark Insights.

[15] Garrido-Trigo A., Corraliza A.M., Veny M., Dotti I., Melón-Ardanaz E., Rill A., Crowell H.L., Corbí Á., Gudiño V., Esteller M. (2023). Macrophage and Neutrophil Heterogeneity at Single-Cell Spatial Resolution in Human Inflammatory Bowel Disease. Nat. Commun..

[16] Jardé T., Chan W.H., Rossello F.J., Kaur Kahlon T., Theocharous M., Kurian Arackal T., Flores T., Giraud M., Richards E., Chan E. (2020). Mesenchymal Niche-Derived Neuregulin-1 Drives Intestinal Stem Cell Proliferation and Regeneration of Damaged Epithelium. Cell Stem Cell.

[17] Cox C.B., Storm E.E., Kapoor V.N., Chavarria-Smith J., Lin D.L., Wang L., Li Y., Kljavin N., Ota N., Bainbridge T.W. (2021). IL-1R1–Dependent Signaling Coordinates Epithelial Regeneration in Response to Intestinal Damage. Sci. Immunol..

[18] Boraschi D., Italiani P., Weil S., Martin M.U. (2018). The Family of the Interleukin-1 Receptors. Immunol. Rev..

[19] Stark R., Grzelak M., Hadfield J. (2019). RNA Sequencing: The Teenage Years. Nat. Rev. Genet..

[20] Liu C.Y., Girish N., Gomez M.L., Kalski M., Bernard J.K., Simons B.D., Polk D.B. (2023). Wound-Healing Plasticity Enables Clonal Expansion of Founder Progenitor Cells in Colitis. Dev. Cell.

[21] Elyada E., Bolisetty M., Laise P., Flynn W.F., Courtois E.T., Burkhart R.A., Teinor J.A., Belleau P., Biffi G., Lucito M.S. (2019). Cross-Species Single-Cell Analysis of Pancreatic Ductal Adenocarcinoma Reveals Antigen-Presenting Cancer-Associated Fibroblasts. Cancer Discov..

[22] Buechler M.B., Pradhan R.N., Krishnamurty A.T., Cox C., Calviello A.K., Wang A.W., Yang Y.A., Tam L., Caothien R., Roose-Girma M. (2021). Cross-Tissue Organization of the Fibroblast Lineage. Nature.

[23] Jasso G.J., Jaiswal A., Varma M., Laszewski T., Grauel A., Omar A., Silva N., Dranoff G., Porter J.A., Mansfield K. (2022). Colon Stroma Mediates an Inflammation-Driven Fibroblastic Response Controlling Matrix Remodeling and Healing. PLoS Biol..

[24] Guo M., Goudarzi K.M., Abedi S., Pieber M., Sjöberg E., Behnan J., Zhang X.-M., Harris R.A., Bartek J., Lindström M.S. (2021). SFRP2 Induces a Mesenchymal Subtype Transition by Suppression of SOX2 in Glioblastoma. Oncogene.

[25] Mukherjee P.K., Nguyen Q.T., Li J., Zhao S., Christensen S.M., West G.A., Chandra J., Gordon I.O., Lin S., Wang J. (2023). Stricturing Crohn’s Disease Single-Cell RNA Sequencing Reveals Fibroblast Heterogeneity and Intercellular Interactions. Gastroenterology.

[26] Dang C.V. (2012). MYC on the Path to Cancer. Cell.

[27] Wang P., Su J., Wang J., Xie Y., Chen W., Zhong J., Wang Y. (2024). NRF1 Promotes Primordial Germ Cell Development, Proliferation and Survival. Cell Prolif..

[28] Ebright R.Y., Zachariah M.A., Micalizzi D.S., Wittner B.S., Niederhoffer K.L., Nieman L.T., Chirn B., Wiley D.F., Wesley B., Shaw B. (2020). HIF1A Signaling Selectively Supports Proliferation of Breast Cancer in the Brain. Nat. Commun..

[29] Garcia-Rendueles M.E.R., Krishnamoorthy G., Saqcena M., Acuña-Ruiz A., Revilla G., de Stanchina E., Knauf J.A., Lester R., Xu B., Ghossein R.A. (2022). Yap Governs a Lineage-Specific Neuregulin1 Pathway-Driven Adaptive Resistance to RAF Kinase Inhibitors. Mol. Cancer.

[30] Artap S., Manderfield L.J., Smith C.L., Poleshko A., Aghajanian H., See K., Li L., Jain R., Epstein J.A. (2018). Endocardial Hippo Signaling Regulates Myocardial Growth and Cardiogenesis. Dev. Biol..

[31] Magoro T., Dandekar A., Jennelle L.T., Bajaj R., Lipkowitz G., Angelucci A.R., Bessong P.O., Hahn Y.S. (2019). IL-1β/TNF-α/IL-6 Inflammatory Cytokines Promote STAT1-Dependent Induction of CH25H in Zika Virus-Infected Human Macrophages. J. Biol. Chem..

[32] Whitley S.K., Balasubramani A., Zindl C.L., Sen R., Shibata Y., Crawford G.E., Weathington N.M., Hatton R.D., Weaver C.T. (2018). IL-1R Signaling Promotes STAT3 and NF-κB Factor Recruitment to Distal Cis-Regulatory Elements That Regulate Il17a/f Transcription. J. Biol. Chem..

[33] Xie X., Shi Q., Wu P., Zhang X., Kambara H., Su J., Yu H., Park S.-Y., Guo R., Ren Q. (2020). Single-Cell Transcriptome Profiling Reveals Neutrophil Heterogeneity in Homeostasis and Infection. Nat. Immunol..

[34] Vafadarnejad E., Rizzo G., Krampert L., Arampatzi P., Arias-Loza A.-P., Nazzal Y., Rizakou A., Knochenhauer T., Bandi S.R., Nugroho V.A. (2020). Dynamics of Cardiac Neutrophil Diversity in Murine Myocardial Infarction. Circ. Res..

[35] Hilligan K.L., Ronchese F. (2020). Antigen Presentation by Dendritic Cells and Their Instruction of CD4+ T Helper Cell Responses. Cell Mol. Immunol..

[36] Gallego C., Vétillard M., Calmette J., Roriz M., Marin-Esteban V., Evrard M., Aknin M.-L., Pionnier N., Lefrançois M., Mercier-Nomé F. (2021). CXCR4 Signaling Controls Dendritic Cell Location and Activation at Steady State and in Inflammation. Blood.

[37] Swiecki M., Wang Y., Riboldi E., Kim A.H.J., Dzutsev A., Gilfillan S., Vermi W., Ruedl C., Trinchieri G., Colonna M. (2014). Cell Depletion in Mice That Express Diphtheria Toxin Receptor under the Control of SiglecH Encompasses More than Plasmacytoid Dendritic Cells. J. Immunol..

[38] Cheng S., Li Z., Gao R., Xing B., Gao Y., Yang Y., Qin S., Zhang L., Ouyang H., Du P. (2021). A Pan-Cancer Single-Cell Transcriptional Atlas of Tumor Infiltrating Myeloid Cells. Cell.

[39] Girish N., Liu C.Y., Gadeock S., Gomez M.L., Huang Y., Sharifkhodaei Z., Washington M.K., Polk D.B. (2021). Persistence of Lgr5+ Colonic Epithelial Stem Cells in Mouse Models of Inflammatory Bowel Disease. Am. J. Physiol. Gastrointest. Liver Physiol..

[40] Ohara T.E., Colonna M., Stappenbeck T.S. (2022). Adaptive Differentiation Promotes Intestinal Villus Recovery. Dev. Cell.

[41] Lee P., Gund R., Dutta A., Pincha N., Rana I., Ghosh S., Witherden D., Kandyba E., MacLeod A., Kobielak K. (2017). Stimulation of Hair Follicle Stem Cell Proliferation through an IL-1 Dependent Activation of γδT-Cells. eLife.

[42] Katsura H., Kobayashi Y., Tata P.R., Hogan B.L.M. (2019). IL-1 and TNFα Contribute to the Inflammatory Niche to Enhance Alveolar Regeneration. Stem Cell Rep..

[43] Patankar J.V., Becker C. (2020). Cell Death in the Gut Epithelium and Implications for Chronic Inflammation. Nat. Rev. Gastroenterol. Hepatol..

[44] Zhu G., Hu J., Xi R. (2021). The Cellular Niche for Intestinal Stem Cells: A Team Effort. Cell Regen..

[45] Talbott H.E., Mascharak S., Griffin M., Wan D.C., Longaker M.T. (2022). Wound Healing, Fibroblast Heterogeneity, and Fibrosis. Cell Stem Cell.

[46] Friedrich M., Pohin M., Jackson M.A., Korsunsky I., Bullers S.J., Rue-Albrecht K., Christoforidou Z., Sathananthan D., Thomas T., Ravindran R. (2021). IL-1-Driven Stromal-Neutrophil Interactions Define a Subset of Patients with Inflammatory Bowel Disease That Does Not Respond to Therapies. Nat. Med..

[47] Koncina E., Nurmik M., Pozdeev V.I., Gilson C., Tsenkova M., Begaj R., Stang S., Gaigneaux A., Weindorfer C., Rodriguez F. (2023). IL1R1+ Cancer-Associated Fibroblasts Drive Tumor Development and Immunosuppression in Colorectal Cancer. Nat. Commun..

[48] Nicolas A.M., Pesic M., Engel E., Ziegler P.K., Diefenhardt M., Kennel K.B., Buettner F., Conche C., Petrocelli V., Elwakeel E. (2022). Inflammatory Fibroblasts Mediate Resistance to Neoadjuvant Therapy in Rectal Cancer. Cancer Cell.

[49] Stuart T., Butler A., Hoffman P., Hafemeister C., Papalexi E., Mauck W.M., Hao Y., Stoeckius M., Smibert P., Satija R. (2019). Comprehensive Integration of Single-Cell Data. Cell.

[50] McGinnis C.S., Murrow L.M., Gartner Z.J. (2019). DoubletFinder: Doublet Detection in Single-Cell RNA Sequencing Data Using Artificial Nearest Neighbors. Cell Syst..

[51] Fenton C.G., Taman H., Florholmen J., Sørbye S.W., Paulssen R.H. (2021). Transcriptional Signatures That Define Ulcerative Colitis in Remission. Inflamm. Bowel Dis..

[52] Zhang L., Yu X., Zheng L., Zhang Y., Li Y., Fang Q., Gao R., Kang B., Zhang Q., Huang J.Y. (2018). Lineage Tracking Reveals Dynamic Relationships of T Cells in Colorectal Cancer. Nature.

[53] Gulati G.S., Sikandar S.S., Wesche D.J., Manjunath A., Bharadwaj A., Berger M.J., Ilagan F., Kuo A.H., Hsieh R.W., Cai S. (2020). Single-Cell Transcriptional Diversity Is a Hallmark of Developmental Potential. Science.

[54] Qiu X., Mao Q., Tang Y., Wang L., Chawla R., Pliner H.A., Trapnell C. (2017). Reversed Graph Embedding Resolves Complex Single-Cell Trajectories. Nat. Methods.

[55] Garcia-Alonso L., Holland C.H., Ibrahim M.M., Turei D., Saez-Rodriguez J. (2019). Benchmark and Integration of Resources for the Estimation of Human Transcription Factor Activities. Genome Res..

[56] Jin S., Guerrero-Juarez C.F., Zhang L., Chang I., Ramos R., Kuan C.-H., Myung P., Plikus M.V., Nie Q. (2021). Inference and Analysis of Cell-Cell Communication Using CellChat. Nat. Commun..

[57] Cooper H.S., Murthy S.N., Shah R.S., Sedergran D.J. (1993). Clinicopathologic Study of Dextran Sulfate Sodium Experimental Murine Colitis. Lab. Investig..

[58] Arstila T., Arstila T.P., Calbo S., Selz F., Malassis-Seris M., Vassalli P., Kourilsky P., Guy-Grand D. (2000). Identical T Cell Clones Are Located within the Mouse Gut Epithelium and Lamina Propia and Circulate in the Thoracic Duct Lymph. J. Exp. Med..

[59] Obermeier F., Dunger N., Deml L., Herfarth H., Schölmerich J., Falk W. (2002). CpG Motifs of Bacterial DNA Exacerbate Colitis of Dextran Sulfate Sodium-Treated Mice. Eur. J. Immunol..

