# Supplementary material for: Myeloid Cell-Derived IL-1 Signaling Damps Neuregulin-1 from Fibroblasts to Suppress Colitis-Induced Early Repair of the Intestinal Epithelium
Source: Int J Mol Sci. 2024 Apr 18;25(8):4469. doi: 10.3390/ijms25084469 (PMC11050633; doi:10.3390/ijms25084469)
Supplement: Supplementary file 1 [file ijms-25-04469-s001.zip › ijms-2942193-supplementary.pdf]

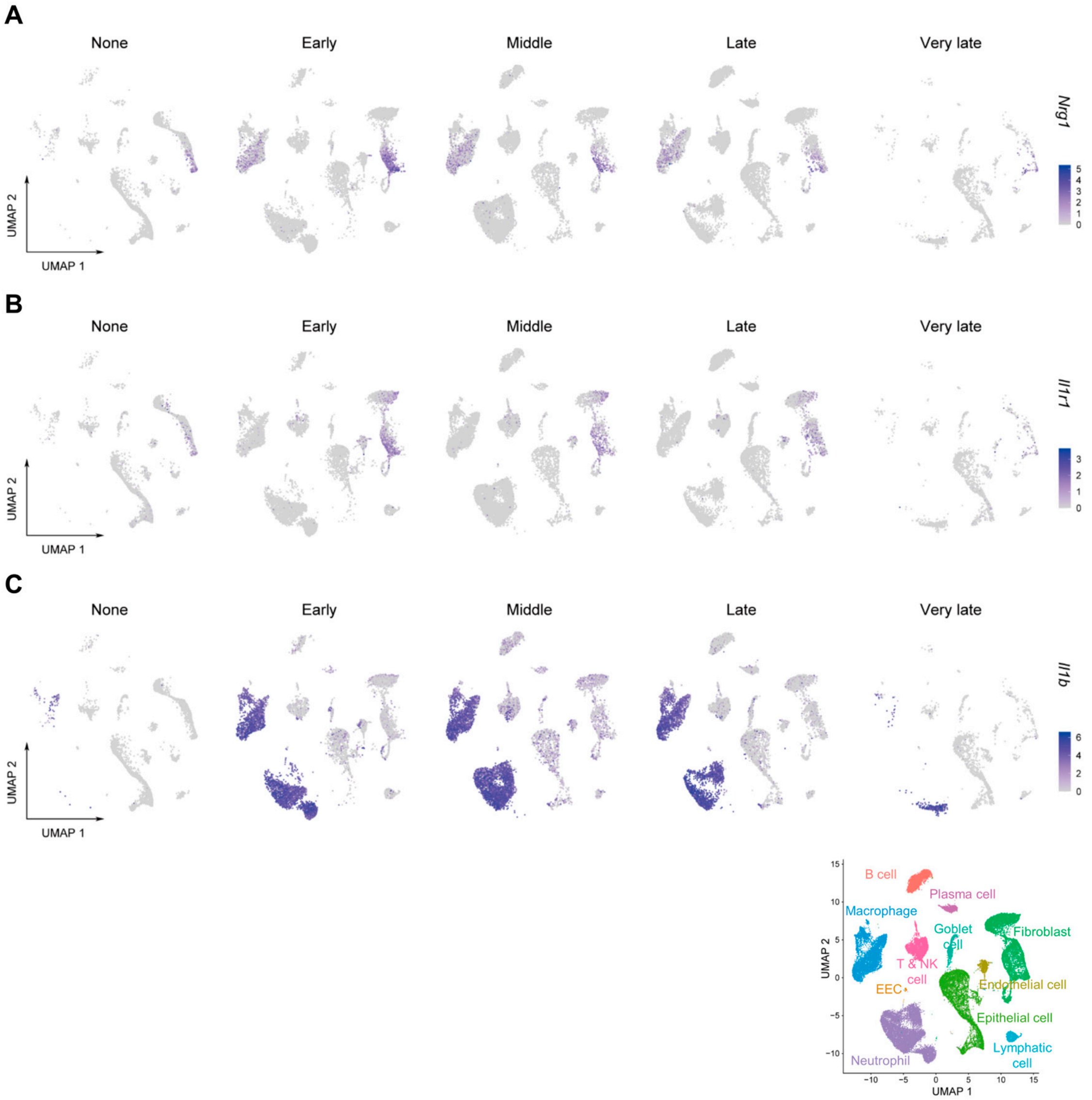

Figure S1. related to Figure 1-4. Spatial expression of *Nrg1*, *Il1r1*, and *Il1b* across cell types at different time points of DSS-colitis. (A-C) UMAP plots depicting the expression patterns of (A) *Nrg1*, (B) *Il1r1*, and (C) *Il1b* in various cell populations at D0, D6, D12, and D18 by scRNA-seq analysis.

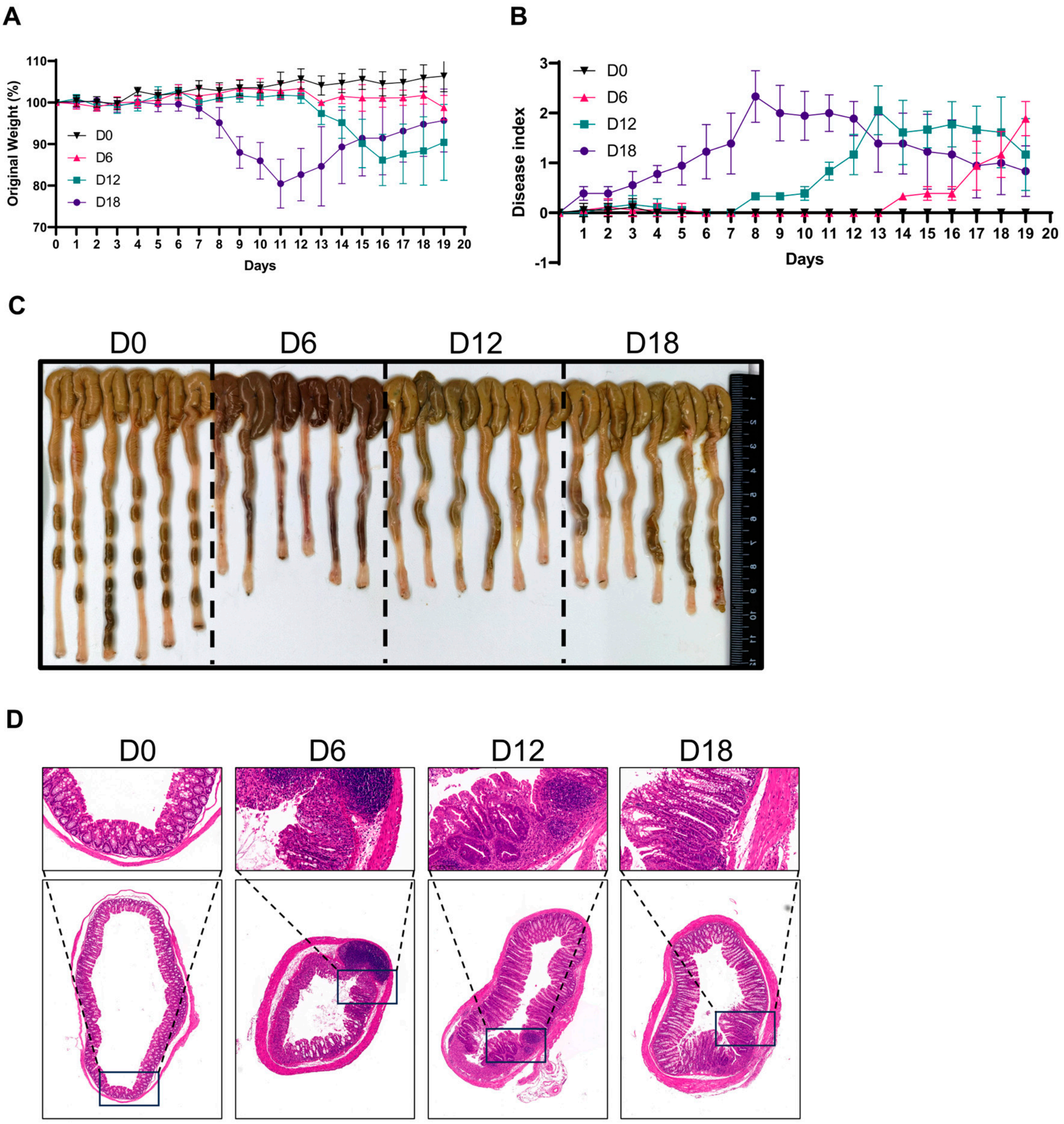

Figure S2. related to Figure 5. DSS induces colitis symptoms with subsequent recovery. (A) Body weight was measured daily. (B) Disease activity index (DAI) scores were monitored daily. (C) Images of colons from control (D0) and DSS-treated mice (D6, D12, D18). (D) Hematoxylin and eosin (H&E) staining of colon tissue sections at 20x magnification. Scale bar represents 100 μm. Data in (A-D) are representative of two independent experiments. Data in (A) are shown as mean ± SD.

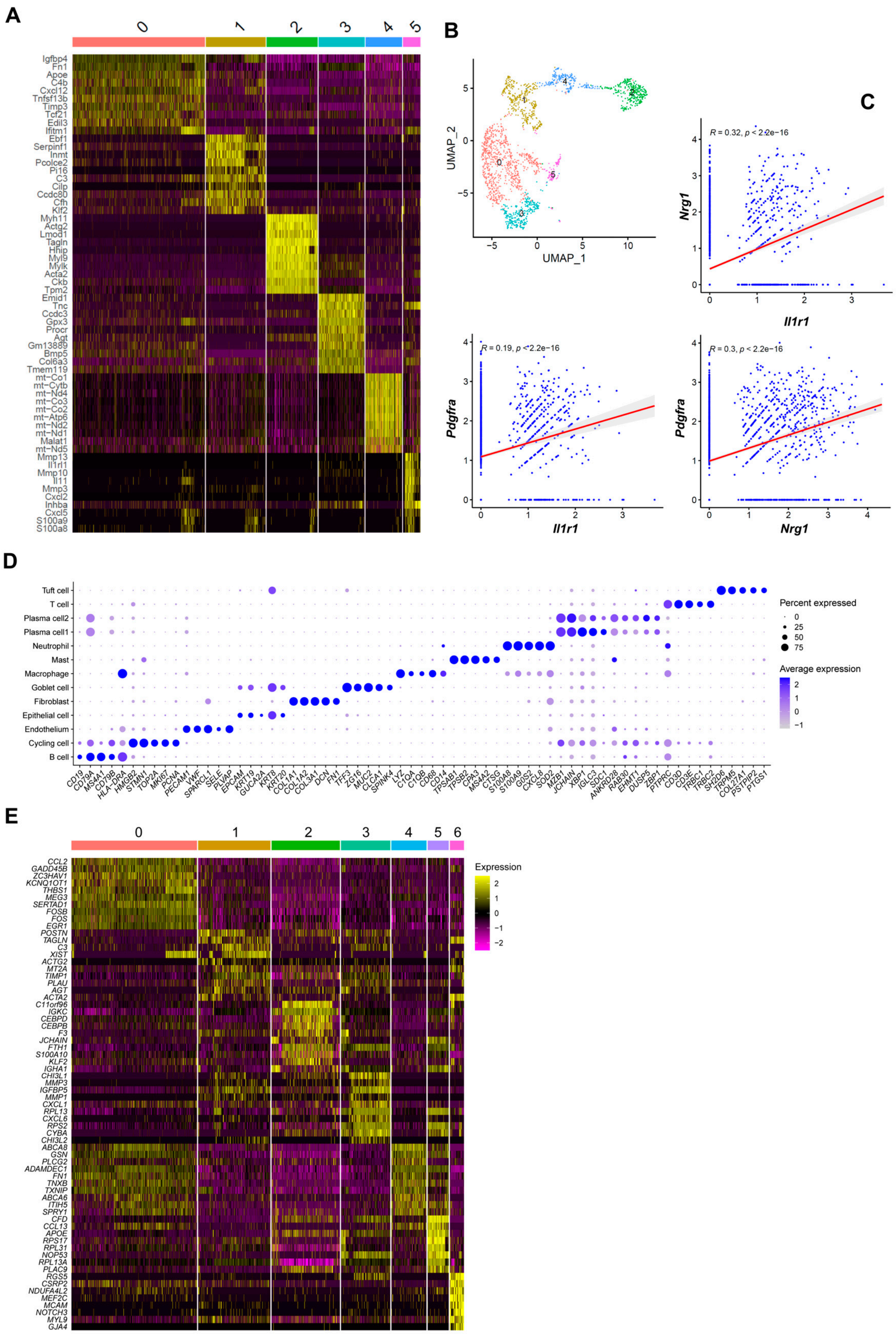

Figure S3. related to Figure 6-7. Expression of Nrg1 and Il1r1 co-located in intestinal fibroblast are positively correlated with each other. (A-C) Single cell RNA-sequencing analysis of colon cells from mice treated with DSS was carried out using the dataset from GEO public database (GSE168033), and heatmap depicting the top 10 most variable genes across six identified fibroblast clusters (A), UMAP projection of these identified fibroblast populations, colored by

cluster affiliation (B), and correlation scatter plots for *Nrg1*, *Il1r1*, and *Pdgfra* expression correlation in whole fibroblasts, with each dot representing a single cell (C) were shown. (D, E) Related to Fig.7, the scRNA-seq data of colonic tissues from human healthy (n=6) and UC (n=6) cohorts was acquired from GSE214695, and bubble plot for the expression level of selected canonical markers in each celltype (D) and heatmap of top 10 highly variable markers for 7 clusters of fibroblasts (E) were shown.
