# Supplementary material for: Myeloid Cell-Derived IL-1 Signaling Damps Neuregulin-1 from Fibroblasts to Suppress Colitis-Induced Early Repair of the Intestinal Epithelium
Source: Int J Mol Sci. 2024 Apr 18;25(8):4469. doi: 10.3390/ijms25084469 (PMC11050633; doi:10.3390/ijms25084469)
Supplement: Supplementary file 1 [file ijms-25-04469-s001.zip › Supplementary Table S1.pdf]

Table S1. Primer sequences

Pdgfra	F	CAAGCAGTGTGCTCCCTAAGT
	R	CACCGGGCAAGCAATATAC
Il1r1	F	GTGCTACTGGGGCTCATTTGT
	R	GGAGTAAGAGGACACTTGCGAAT
Nrg1	F	ATGGAGATTTATCCCCCAGACA
	R	GTTGAGGCACCCTCTGAGAC
Gapdh	F	TTCACCACCATGGAGAAGGC
	R	GGCATGGACTGTGGTCATGA
